# Supplementary material for: Efficacy of Different Bacillus of Calmette-Guérin (BCG) Strains on Recurrence Rates among Intermediate/High-Risk Non-Muscle Invasive Bladder Cancers (NMIBCs): Single-Arm Study Systematic Review, Cumulative and Network Meta-Analysis
Source: Cancers (Basel). 2023 Mar 23;15(7):1937. doi: 10.3390/cancers15071937 (PMC10093360; doi:10.3390/cancers15071937)
Supplement: Supplementary file 1 [file cancers-15-01937-s001.zip › cancers-2191263-supplementary.pdf]

Supplementary Materials: Efficacy of different Bacillus of Calmette-Guérin (BCG) Strains on Recurrence Rates among Intermediate/High-risk Non-muscle Invasive Bladder Cancers (NMIBCs): Single-arm Study Systematic Review, Cumulative and Network Meta-analysis

Francesco Del Giudice, Vincenzo Asero, Eugenio Bologna, Carlo Maria Scornajenghi, Dalila Carino, Virginia Dolci, Pietro Viscuso, Stefano Salciccia, Alessandro Sciarra, David D'Andrea, Benjamin Pradere, Marco Moschini, Andrea Mari, Simone Albisinni, Wojciech Krajewski, Ekaterina Laukhtina, Andrea Gallioli, Laura S. Mertens, Gautier Marcq, Alessia Cimadamore, Luca Afferi, Francesco Soria, Keiichiro Mori, Karl Tuly, Renate Pichler, Matteo Ferro, Tataru Octavian Sabin, Riccardo Autorino, Simone Crivellaro, Felice Crocetto, Satvir Basran, Michael L. Eisenberg, Benjamin I. Chung and Ettore De Berardinis ***- on behalf of the European Association of Urology - Young Academic Urologists (EAU-YAU): Urothelial Carcinomas Working Group***

Table S1. Comprehensive list of primary and secondary search criteria fields. **NMIBC**: Non-Muscle Invasive Bladder Cancer; **BCG:** Bacillus Calmette–Guérin; **CHT**: Chemotherapy; **TURB**: Transurethral resection of the bladder.

List of search terms	
**Primary fields**	**Secondary fields**
bladder cancer	Transurethral resection (TURBT)
NMIBC	Transurethral resection w/o intravesical BCG
BCG	Transurethral resection w/o intravesical chemotherapy
BCG strains	Transurethral resection w/o intravesical BCG (Either different strain or different dose)
BCG Connaught	Transurethral resection w/o a non-BCG biological (Defined as either interferon-α2b)
BCG Tice	BCG strain comparison
BCG Tokyo 172	Transurethral resection w/o a combination of intravesical treatments
BCG RIVM	BCG and/or Intravescical CHT
BCG Pasteur	Mitomycin-C (MMC)
BCG Danish 1131	Adjuvant combined intravesical therapy
Sii Onco BCG	BCG optimal schedule
BCG Moreau	BCG optimal dose
BCG Glaxo	Follow-up
BCG Montreal Armand Frappier	

Table S2. Risk assessment of individual study enrolled according to the *"Quality assessment tool for observational Cohort and Cross-Sectional Studies"* released by the National Health Institute (NIH). **NA**: Not applicable.

Author	year	Criteria 1	Criteria 2	Criteria 3	Criteria 4	Criteria 5	Criteria 6	Criteria 7	Criteria 8	Criteria 9	Criteria 10	Criteria 11	Criteria 12	Criteria 13	Criteria 14
Agrawal [23]	2007	+	+	+	+	-	+	+	NA	NA	NA	+	NA	NA	+
Akaza [24]	1995	+	+	+	+	-	+	+	NA	NA	NA	+	NA	NA	+
Akaza [25]	2003	+	+	+	+	+	+	-	NA	NA	NA	+	NA	NA	+
Ali-el-dein [26]	1999	+	+	+	+	-	+	+	NA	NA	NA	+	NA	NA	+
Arend [27]	2016	+	+	-	+	+	+	-	NA	NA	NA	+	NA	NA	+
Bilen [28]	2000	+	+	+	+	-	+	-	NA	NA	NA	+	NA	NA	+
Brosman [29]	1982	+	+	+	+	+	+	-	NA	NA	NA	+	NA	NA	+
Cai [30]	2008	+	+	+	+	+	+	-	NA	NA	NA	+	NA	NA	+
Cheng [31]	2005	+	+	+	+	+	+	+	NA	NA	NA	+	NA	NA	+
D'Andrea [32]	2020	+	+	+	+	+	+	+	NA	NA	NA	+	NA	NA	+
Dai Koguchi [33]	2020	+	+	+	+	+	+	+	NA	NA	NA	+	NA	NA	+
Del Giudice [34]	2021	+	+	+	+	-	+	+	NA	NA	NA	+	NA	NA	+
Del Giudice [35]	2022	+	+	+	+	+	+	+	NA	NA	NA	+	NA	NA	+
Dereijke [36]	2005	+	+	-	+	+	+	+	NA	NA	NA	+	NA	NA	+
Di Lorenzo [37]	2010	+	+	+	+	+	+	-	NA	NA	NA	+	NA	NA	+
Di Stasi [38]	2006	+	+	+	+	+	+	+	NA	NA	NA	+	NA	NA	+
Farah [39]	2014	+	+	+	+	+	+	+	NA	NA	NA	+	NA	NA	+
Friedrich [40]	2007	+	+	+	+	-	+	+	NA	NA	NA	+	NA	NA	+
Gontero [41]	2013	+	+	-	+	-	+	-	NA	NA	NA	+	NA	NA	+
Gruenwald [42]	1997	+	+	+	+	+	+	-	NA	NA	NA	+	NA	NA	+
Hemdan [43]	2014	+	+	+	+	-	+	+	NA	NA	NA	+	NA	NA	+
Herr [44]	2012	+	+	+	+	+	+	-	NA	NA	NA	+	NA	NA	+
Herr [45]	2011	+	+	+	+	-	+	+	NA	NA	NA	+	NA	NA	+
Herr [46]	2007	+	+	+	+	+	+	+	NA	NA	NA	+	NA	NA	+
Hinotsu [47]	2011	+	+	+	+	+	+	-	NA	NA	NA	+	NA	NA	+
Hinotsu [48]	2006	+	+	+	+	+	+	+	NA	NA	NA	+	NA	NA	+
Hudson [49]	1987	+	+	+	+	+	+	-	NA	NA	NA	+	NA	NA	+
Ibrahiem [50]	1988	+	+	+	+	+	+	-	NA	NA	NA	+	NA	NA	+
Inamoto [51]	2013	+	+	+	+	-	+	-	NA	NA	NA	+	NA	NA	+
Jarvien [52]	2009	+	+	+	+	+	+	+	NA	NA	NA	+	NA	NA	+
Kamat [53]	1994	+	+	-	+	+	+	+	NA	NA	NA	+	NA	NA	+
Koga [54]	2010	+	+	+	-	+	+	-	NA	NA	NA	+	NA	NA	+
Lamm [2]	2000	+	+	-	+	+	+	-	NA	NA	NA	+	NA	NA	+
Lamm [55]	1995	+	+	-	+	+	+	+	NA	NA	NA	+	NA	NA	+
Martinez-Pieniro [56]	2002	+	+	+	+	-	+	+	NA	NA	NA	+	NA	NA	+
Martinez-Pieniro [57]	1990	+	+	+	+	-	+	+	NA	NA	NA	+	NA	NA	+
Marttila [58]	2016	+	+	+	+	+	+	+	NA	NA	NA	+	NA	NA	+
Melekos [59]	1996	+	+	+	+	-	+	+	NA	NA	NA	+	NA	NA	+
Mukherjee [60]	1992	+	+	+	+	+	+	+	NA	NA	NA	+	NA	NA	+
Nowak [61]	2021	+	+	+	+	-	+	+	NA	NA	NA	+	NA	NA	+
Oddens [3]	2012	+	+	+	+	+	+	+	NA	NA	NA	+	NA	NA	+
Ojea [62]	2007	+	+	-	-	+	+	+	NA	NA	NA	+	NA	NA	+
Okamura [63]	2011	+	+	+	+	+	+	+	NA	NA	NA	+	NA	NA	+
Oosterlinck [64]	2011	+	+	+	+	+	+	+	NA	NA	NA	+	NA	NA	+
Ourfali [65]	2019	+	+	+	+	+	+	-	NA	NA	NA	+	NA	NA	+
Palou [66]	2001	+	+	+	+	-	+	+	NA	NA	NA	+	NA	NA	+
Peyromaure [83]	2003	+	+	+	+	+	+	+	NA	NA	NA	+	NA	NA	+
Porena [67]	2010	+	+	-	+	+	+	+	NA	NA	NA	+	NA	NA	+
Prasanna [68]	2017	+	+	+	+	+	+	-	NA	NA	NA	+	NA	NA	+
Rentsch [69]	2014	+	+	+	+	+	+	+	NA	NA	NA	+	NA	NA	+
Rintala [70]	1991	+	+	+	+	+	+	-	NA	NA	NA	+	NA	NA	+
Sekine [71]	2001	+	+	-	-	-	+	+	NA	NA	NA	+	NA	NA	+
Sengiku [72]	2013	+	+	+	+	-	+	+	NA	NA	NA	+	NA	NA	+
Shinka [73]	1997	+	+	+	+	+	+	-	NA	NA	NA	+	NA	NA	+
Shinka [74]	1989	+	+	+	+	-	+	-	NA	NA	NA	+	NA	NA	+
Sood [75]	2020	+	+	+	+	+	+	+	NA	NA	NA	+	NA	NA	+
Steinberg [76]	2016	+	+	+	+	-	+	-	NA	NA	NA	+	NA	NA	+
Sylvester [77]	2010	+	+	+	+	+	+	+	NA	NA	NA	+	NA	NA	+

Takashi [78]	1998	+	+	+	+	+	+	+	NA	NA	NA	+	NA	NA	+
Takashi [79]	1997	+	+	+	+	+	+	+	NA	NA	NA	+	NA	NA	+
Witjes [80]	2016	+	+	+	+	+	+	+	NA	NA	NA	+	NA	NA	+
Witjes [81]	1999	+	+	+	-	+	+	+	NA	NA	NA	+	NA	NA	+
Yalcinkaya [82]	1997	+	+	-	+	-	+	+	NA	NA	NA	+	NA	NA	+
Yoo [84]	2012	+	+	+	+	+	+	-	NA	NA	NA	+	NA	NA	+

NA: not applicable. Criteria 1: Was the research question or objective in this paper clearly stated? Criteria 2: Was the study population clearly specified and defined? Criteria 3: Was the participation rate of eligible persons at least 50%? Criteria 4: Were all the subjects selected or recruited from the same or similar populations (including the same time period)? Were inclusion and exclusion criteria for being in the study prespecified and applied uniformly to all participants? Criteria 5: Were a sample size justification, power description, or variance and effect estimates provided? Criteria 6: For the analyses in this paper, was the exposure(s) of interest measured prior to the outcome(s) being measured? Criteria 7: Was the timeframe sufficient so that one could reasonably expect to see an association between exposure and outcome if it existed? Criteria 8: For exposures that can vary in amount or level, did the study examine different levels of the exposure as related to the outcome? Criteria 9: Were the exposure measures (independent variables) clearly defined, valid, reliable, and implemented consistently across all study participants? Criteria 10: Was the exposure(s) assessed more than once over time? Criteria 11: Were the outcome measures (dependent variables) clearly defined, valid, reliable, and implemented consistently across all study participants? Criteria 12: Were the outcome assessors blinded to the exposure status of participants? Criteria 13: Was loss to follow-up after baseline 20% or less? Criteria 14: Were key potential confounding variables measured and adjusted statistically for their impact on the relationship between exposure(s) and outcome(s)?

Table S3. Multi-treatment level comparison (OR, 95%CI) for the risk of BC recurrence among the n=7 available BCG stains assessed in the network meta-analysis (BCG Tice considered as the reference standard). **BCG:** Bacillus Calmette–Guérin; **OR**: odd ratio; **CI**: Confidence Interval; **SE**: standard error.

BCG Strain	OR	95% CI Lower	95% CI Upper	SE	z	P>|z|
Tice	Ref.	-	-	-	-	-
Connaught	0.75	0.53	1.07	0.18	-1.58	0.114
Glaxo	0.74	0.09	5.92	1.07	-0.29	0.773
Moreau	0.69	0.43	1.12	0.25	-1.5	0.135
Pasteur	0.66	0.24	1.76	0.50	-0.84	0.403
RIVM	1.07	0.72	1.58	0.20	0.32	0.748
Tokyo 172	0.91	0.38	2.16	0.44	-0.21	0.831

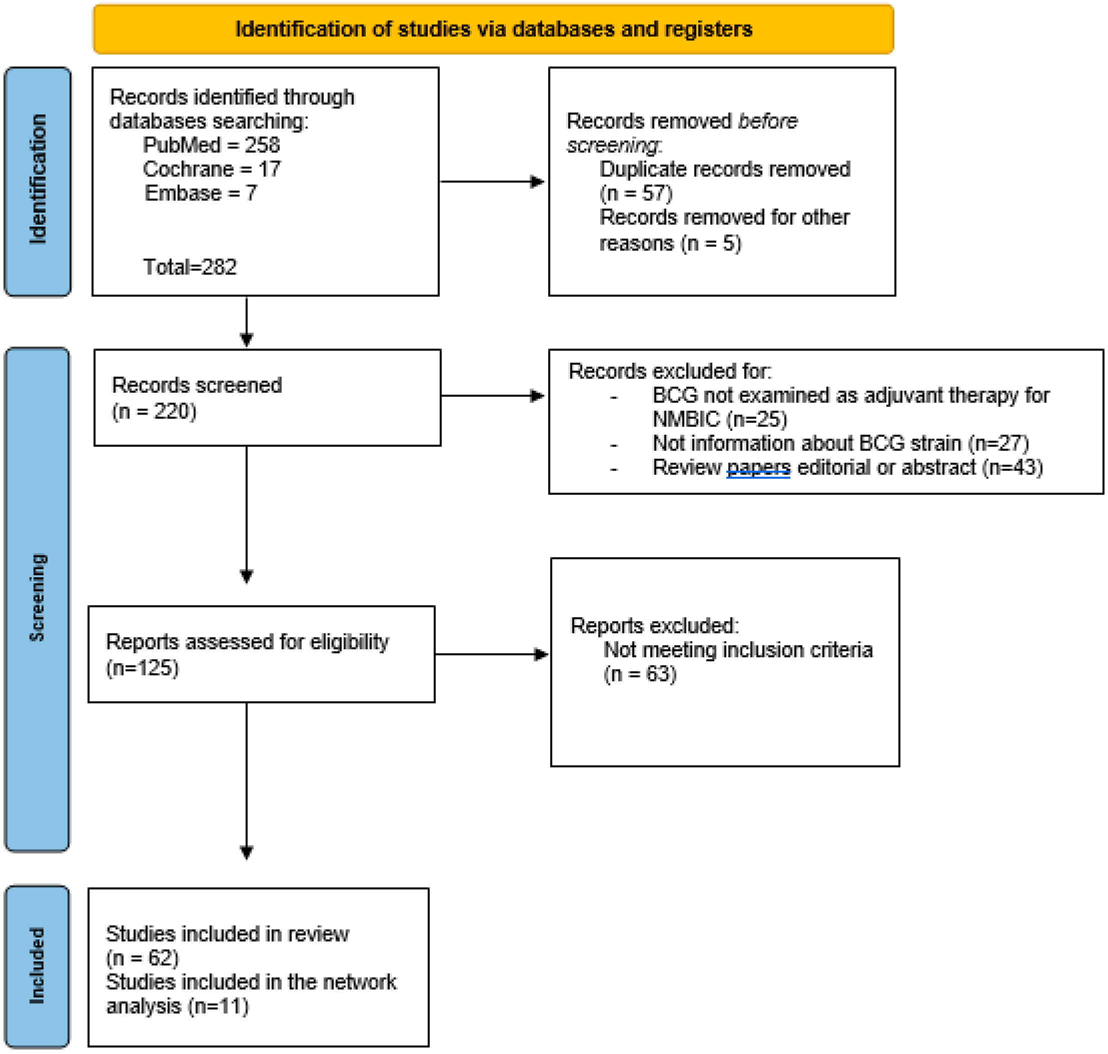

Figure S1. PRISMA flow diagram. **NMIBC**: Non-Muscle Invasive Bladder Cancer; **BCG:** Bacillus Calmette–Guérin.

A

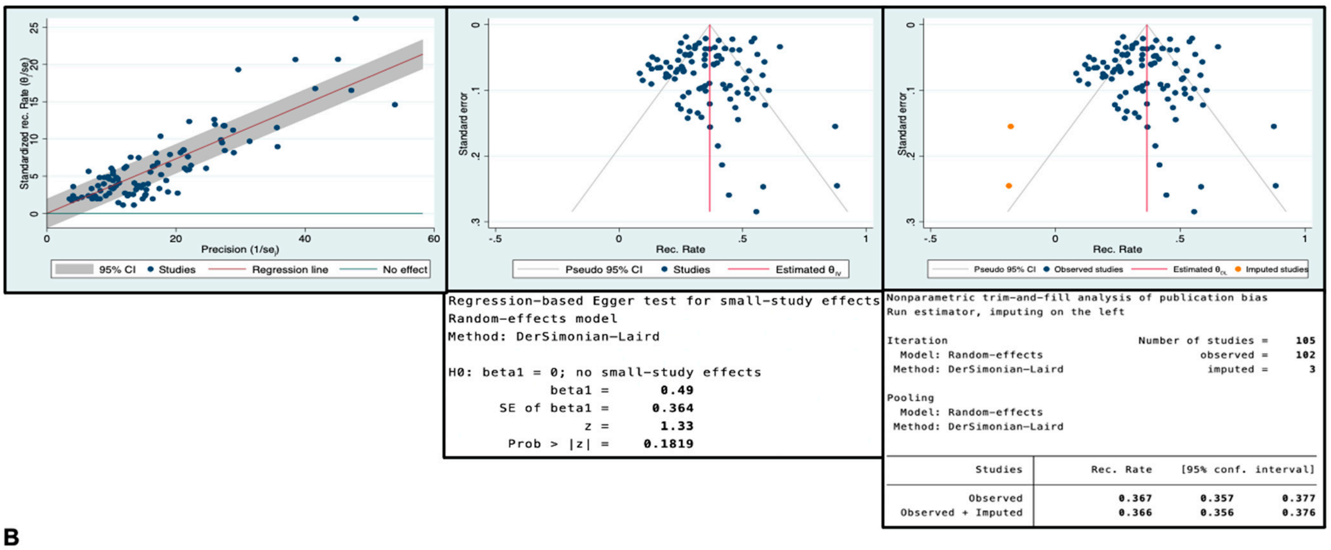


```
Regression-based Egger test for small-study effects
Random-effects model
Method: DerSimonian-Laird

H0: beta1 = 0; no small-study effects
            beta1 =        0.49
      SE of beta1 =       0.364
                z =        1.33
       Prob > |z| =      0.1819
```

```
Nonparametric trim-and-fill analysis of publication bias
Run estimator, imputing on the left

Iteration                          Number of studies =    105
  Model: Random-effects                     observed =    102
 Method: DerSimonian-Laird                   imputed =      3

Pooling
  Model: Random-effects
 Method: DerSimonian-Laird

                   Studies |   Rec. Rate   [95% conf. interval]
---------------------------+-----------------------------------
                  Observed |       0.367       0.357      0.377
       Observed + Imputed  |       0.366       0.356      0.376
```

B

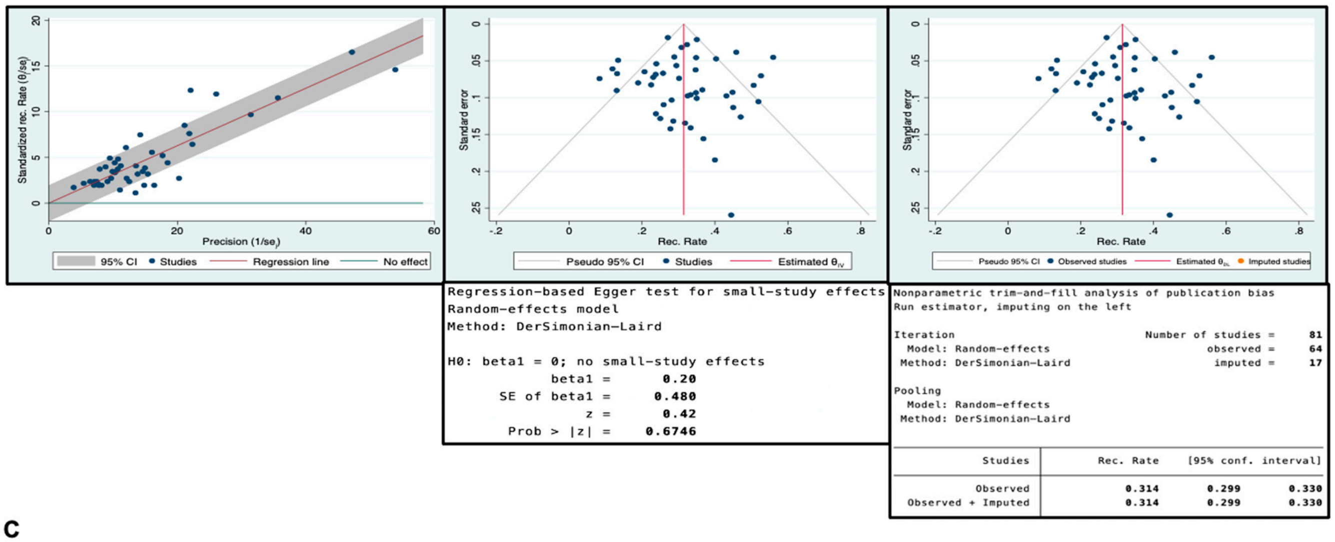


```
Regression-based Egger test for small-study effects
Random-effects model
Method: DerSimonian-Laird

H0: beta1 = 0; no small-study effects
            beta1 =        0.20
      SE of beta1 =       0.480
                z =        0.42
       Prob > |z| =      0.6746
```

```
Nonparametric trim-and-fill analysis of publication bias
Run estimator, imputing on the left

Iteration                          Number of studies =     81
  Model: Random-effects                     observed =     64
 Method: DerSimonian-Laird                   imputed =     17

Pooling
  Model: Random-effects
 Method: DerSimonian-Laird

                   Studies |   Rec. Rate   [95% conf. interval]
---------------------------+-----------------------------------
                  Observed |       0.314       0.299      0.330
       Observed + Imputed  |       0.314       0.299      0.330
```

C

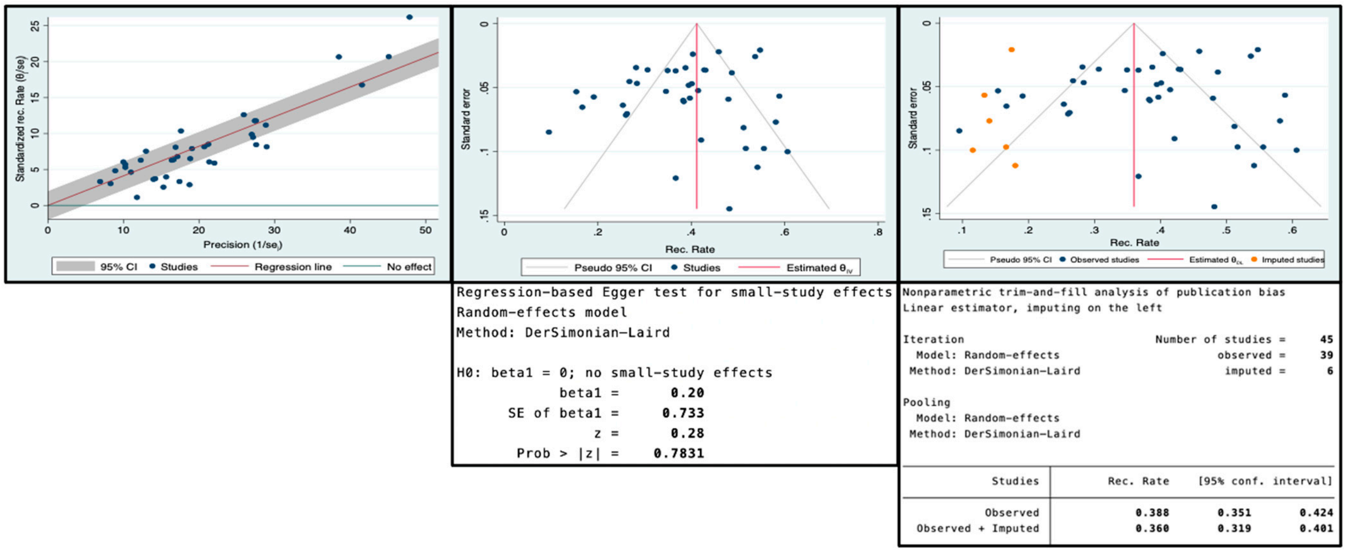


```
Regression-based Egger test for small-study effects
Random-effects model
Method: DerSimonian-Laird

H0: beta1 = 0; no small-study effects
            beta1 =        0.20
      SE of beta1 =       0.733
                z =        0.28
       Prob > |z| =      0.7831
```

```
Nonparametric trim-and-fill analysis of publication bias
Linear estimator, imputing on the left

Iteration                          Number of studies =     45
  Model: Random-effects                     observed =     39
 Method: DerSimonian-Laird                   imputed =      6

Pooling
  Model: Random-effects
 Method: DerSimonian-Laird

                   Studies |   Rec. Rate   [95% conf. interval]
---------------------------+-----------------------------------
                  Observed |       0.388       0.351      0.424
       Observed + Imputed  |       0.360       0.319      0.401
```

Figure S2. Publication bias for recurrence rate across the total studies included expressed by Galbright plot (I), Funnel plot with regression-based Egger-test for small-study effects (II) and Funnel plot after study's imputation with the "Trim and Fill" method (III) (**A**). Publication bias for BC recurrence rate by studies with ≤3-yr RFS endpoints assessed throughout Galbright plot (I), Funnel

with regression-based Egger test for small-study effects (II), Funnel plot after the “Trim and Fill” method (III) (**B**). Publication bias for BC recurrence rate by studies with >3-yr RFS endpoints assessed throughout Galbright plot (I), Funnel with regression-based Egger test for small-study effects (II), Funnel plot after study’s imputation with the “Trim and Fill” method (III) (**C**). **RFS**: Recurrence-free survival; **CI**: Confidence Interval.

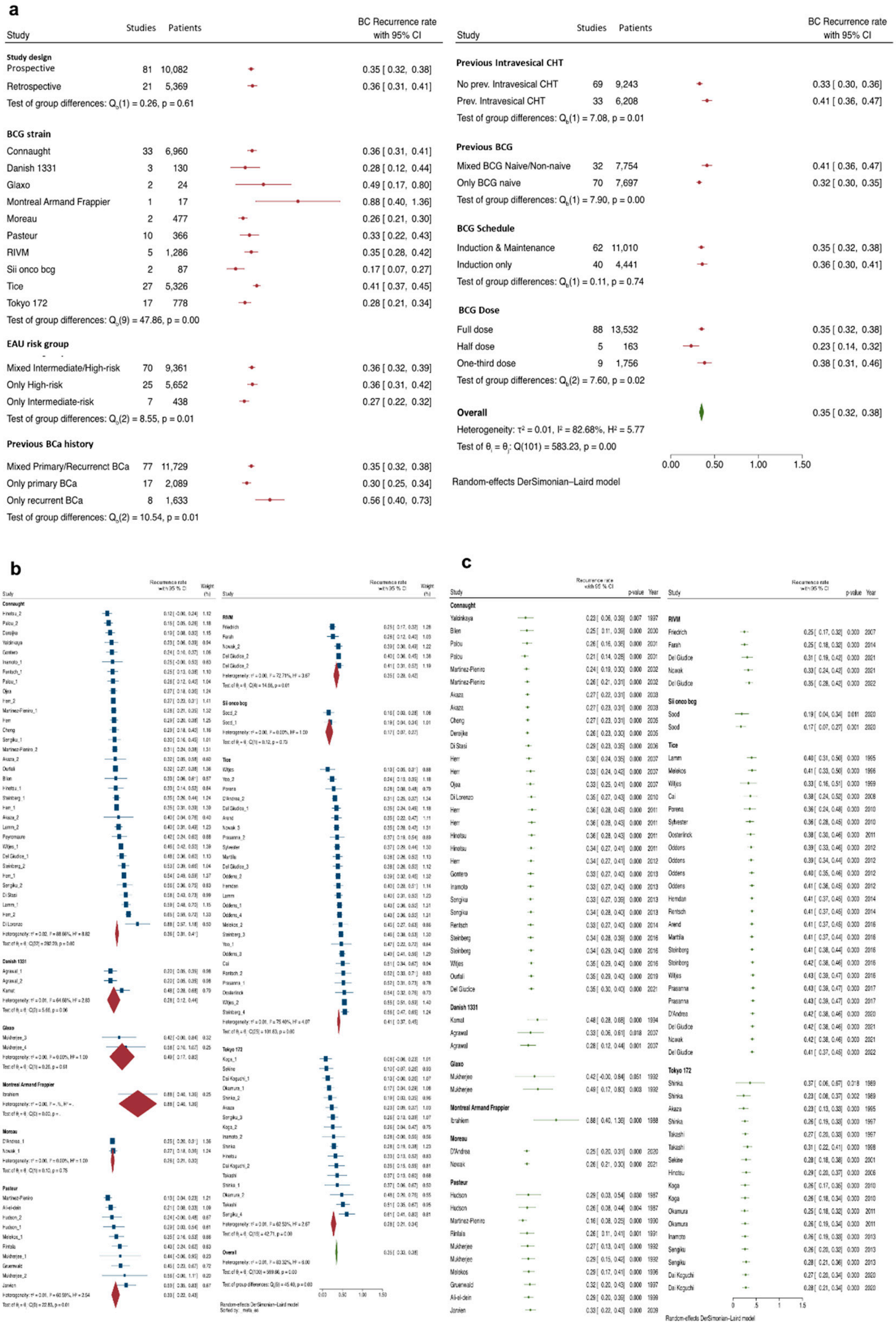

Figure S3. Forrest plot depicting BC recurrence rate sorted by BCG strains for all the studies enrolled in the systematic review and meta-analysis (**A**). Sub-groups analysis exploring heterogeneity according to BC recurrence rate stratified by categorical confounders (e.g., study, BCG and NMIBC

characteristics) (**B**). Forrest-plot for cumulative meta-analysis sorted by publication year and stratified according to BCG strain (**C**). **BC**: Bladder Cancer; **BCG:** Bacillus Calmette–Guérin; **NMIBC**: Non-Muscle Invasive Bladder Cancer; **CHT**: Chemotherapy; **EAU:** European Association of Urology.

A

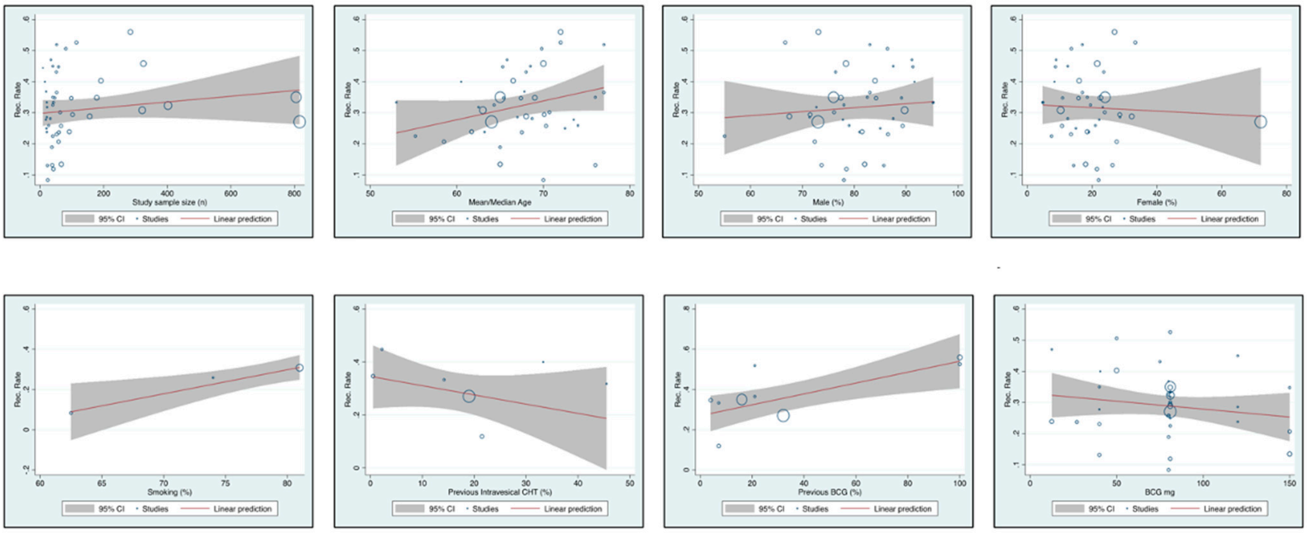

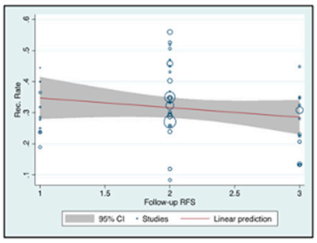

Quantitative Variable	Meta-regression					
	Coef.	*SE*	*Z-statistic*	*P value*	*95% CI*	
**Study sample size (n)**	0.0000	0.0000	1.13	0.258	-0.0000	0.0002
**Median Age**	0.0060	0.0035	1.73	0.084	-0.0008	0.0129
**Male (%)**	0.0012	0.0023	0.55	0.582	-0.0032	0.0058
**Female (%)**	-0.0005	0.0015	-0.36	0.721	-0.0035	0.0024
**Smoking (%)**	0.0119	0.0043	2.77	0.006	0.0035	0.0204
**Previous intravesical CHT (%)**	-0.0034	0.0031	-1.11	0.265	-0.0096	0.0026
**Previous BCG (%)**	0.0027	0.0009	2.90	0.004	0.0008	0.0045
**BCG mg**	-0.0005	0.0005	-1.02	0.309	-0.0014	0.0047
**Follow-up RFS**	-0.0309	0.0268	-1.15	0.248	-0.0835	0.0216

B

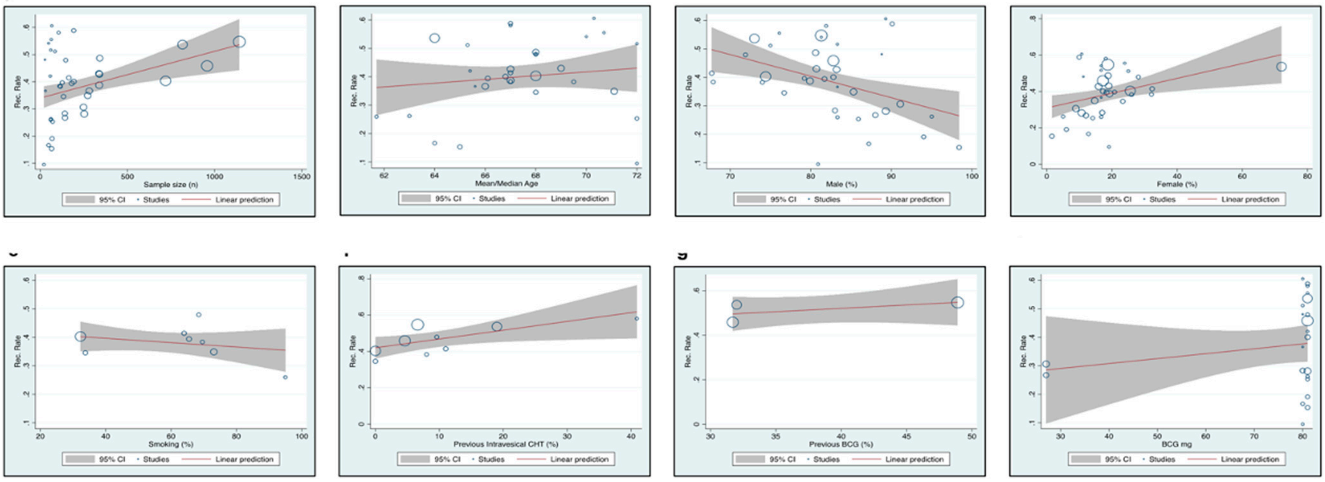

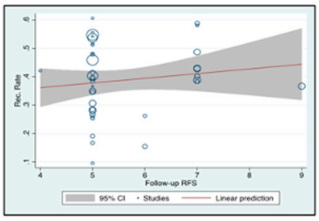

Quantitative Variable	Meta-regression					
	Coef.	*SE*	*Z-statistic*	*P value*	*95% CI*	
**Study sample size (n)**	0.0001	0.0000	3.27	0.00	0.0000	0.0002
**Median Age**	0.0065	0.0080	0.81	0.41	-0.0092	0.0224
**Male (%)**	-0.0075	0.0024	-3.06	0.00	-0.1242	-0.0027
**Female (%)**	0.0040	0.0014	2.74	0.006	0.0011	0.0069
**Smoking (%)**	-0.0007	0.0087	-0.88	0.381	-0.0024	-0.0009
**Previous intravesical CHT (%)**	0.0048	0.0022	2.15	0.032	0.0004	0.0092
**Previous BCG (%)**	0.0030	0.0038	0.79	0.431	-0.0045	0.0105
**BCG mg**	0.0017	0.0018	0.91	0.361	-0.0019	0.0054
**Follow-up RFS**	0.0165	0.0181	0.91	0.363	-0.0190	0.0520

Figure S4. Meta-regression analysis for studies with ≤3-yr RFS endpoint modeling BC recurrence rate according to available continuous demographic, clinic, and BCG-related variables (**A**). Meta-regression analysis for studies with >3-yr RFS endpoint modeling BC recurrence rate according to available continuous demographic, clinic and BCG-related variables (**B**) **BC**: Bladder Cancer; **BCG:** Bacillus Calmette–Guérin; **CHT**: Chemotherapy; **RFS**: Recurrence-free survival.

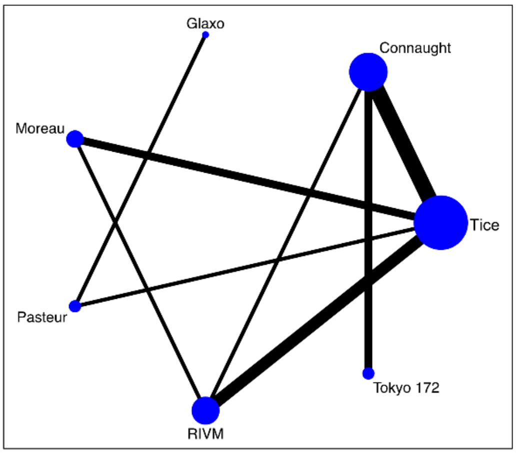

Figure S5. Network map for multiple-treatment comparison out of the n=7 BCG strains included in the analysis.

a

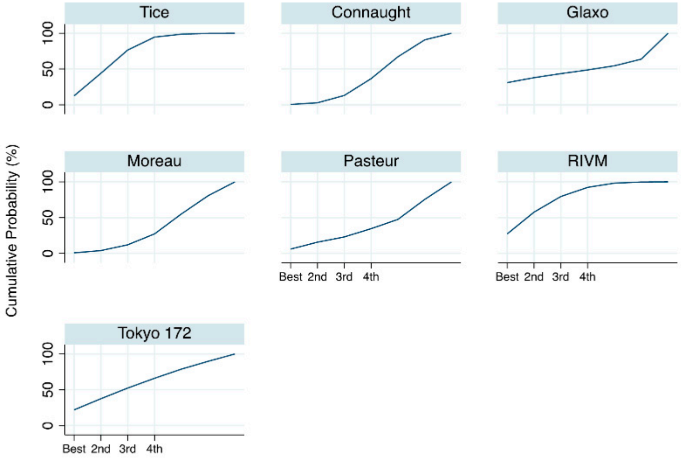

b

Study Rank	Tice	Connaught	Glaxo	Moreau	Pasteur	RIVM	Tokyo 172
Best	12.5	0.5	31.0	0.9	6.0	27.2	21.9
2nd	31.7	2.4	7.0	3.0	9.6	30.6	15.7
3rd	32.4	10.1	5.5	8.1	7.4	21.8	14.7
4th	18.1	23.6	5.2	15.2	11.5	12.6	13.8
5th	4.0	30.5	5.9	27.8	13.0	6.1	12.7
6th	1.2	23.6	9.1	25.7	27.9	1.5	11.0
Worst	0.1	9.3	36.3	19.3	24.6	0.2	10.2

Figure S6. The surface under the cumulative ranking curve (SUCRA) stratified by each BCG strains (**A**) and its related cumulative probability for being the best vs. the worst BCG treatment strain (**B**). **BCG:** Bacillus Calmette–Guérin.
